# Effects of COMT rs4680 and BDNF rs6265 polymorphisms on brain degree centrality in Han Chinese adults who lost their only child

**DOI:** 10.1038/s41398-020-0728-7

**Published:** 2020-01-30

**Authors:** Rongfeng Qi, Yifeng Luo, Li Zhang, Yifei Weng, Wesley Surento, Lingjiang Li, Zhihong Cao, Guang Ming Lu

**Affiliations:** 1 Department of Medical Imaging, Jinling Hospital, Medical School of Nanjing University, Nanjing, Jiangsu 210002 China; 2 Imaging Genetics Center, Mark and Mary Stevens Neuroimaging and Informatics Institute, University of Southern California, Marina del Rey, CA 90292 USA; 3 Department of Radiology, The Affiliated Yixing Hospital of Jiangsu University, Wuxi, 75 Tongzhenguan Road, 214200 Wuxi, China; 4 Key Laboratory of Psychiatry and Mental Health of Hunan Province, Mental Health Institute, The Second Xiangya Hospital, National Technology Institute of Psychiatry, Central South University, Changsha, Hunan 410011 China

**Keywords:** Psychiatric disorders, Neuroscience

## Abstract

Losing one’s only child is a major traumatic life event that may lead to posttraumatic stress disorder (PTSD); however, not all parents who experience this trauma develop PTSD. Genetic variants are associated with the risk of developing PTSD. Catechol-*O*-methyltransferase (*COMT*) rs4680 and brain-derived neurotrophic factor (*BDNF*) rs6265 are two most well-described single-nucleotide polymorphisms that relate to stress response; however, the neural mechanism underlying their effects on adults who lost an only child remains poorly understood. Two hundred and ten Han Chinese adults who had lost their only child (55 with PTSD and 155 without PTSD) were included in this imaging genetics study. Participants were divided into subgroups according to their *COMT* rs4680 and *BDNF* rs6265 genotypes. Degree Centrality (DC)—a resting-state fMRI index reflecting the brain network communication—was compared with a three-way (PTSD diagnosis, *COMT*, and *BDNF* polymorphisms) analysis of covariance. Diagnosis state had a significant effect on DC in bilateral inferior parietal lobules and right middle frontal gyrus (MFG), where PTSD adults showed weaker DC. *BDNF* × diagnosis interaction effect was found in the right MFG and hippocampus, and these two regions were reversely modulated. Also, there was a significant *COMT* × *BDNF* interaction effect in left cuneus, middle temporal gyrus, right inferior occipital gyrus, and bilateral putamen, independent of PTSD diagnosis. These findings suggest that the modulatory effect of *BDNF* polymorphism on the MFG and hippocampus may contribute to PTSD development in bereaved adults. Interactions of *COMT* × *BDNF* polymorphisms modulate some cortices and basal ganglia, irrespective of PTSD development.

## Introduction

The “One-Child Policy”—which permits each family to have only one child—was implemented in mainland China for more than 30 years^1,2^. Although this policy succeeded in slowing the rapid population growth rate in China, its associated problems are also becoming apparent. For example, it has brought about a new phenomenon of “childless” older Chinese adults, these parents lost their only child at a time when they were unable to bear another. This causes them to experience long-term grief, loneliness, anxiety, depression, and posttraumatic stress disorder (PTSD)^3–5^. Although the One-Child Policy ended in 2015, the number of families that might lose their only child will possibly continue to increase for a considerable period of time^6^, which could lead to significant and widespread public health problems.

PTSD is characterized by dysregulated fear conditioning, extinction, and stress responses^7–9^. Functional neuroimaging studies have identified the dysfunction of several brain regions involved in the regulation of fear and stress responses in PTSD, which mainly includes the amygdala, hippocampus, and prefrontal cortex^9–11^. These findings strongly support the prefrontal-limbic imbalance theory in the pathology of PTSD^8,11–13^. It should be noted that not all adults who experience the trauma of losing their only child will go on to develop PTSD^14^. The underlying mechanisms of PTSD development remain inadequately investigated and poorly understood. Twin and heritability studies have consistently suggested that at least one-third of the variance in PTSD risk is determined by genetic components^15–17^. A variety of candidate genes in the dopaminergic system, hypothalamic-pituitary-adrenal axis, serotonergic system, and neuroinflammation have been conducted in PTSD studies^18–20^. Also, several unbiased genome-wide association studies (GWAS) based on the clinical diagnosis of PTSD have been performed^21–24^. These studies have helped us to understand the genetic basis of PTSD development and its genetic overlap with other mental disorders such as schizophrenia^24^. However, prior candidate-gene studies and even GWAS have largely been unsuccessful at determining an overlapped or replicable risk gene for PTSD^19^. The failure to replicate these results could possibly be due to the wide variations in the types of trauma, the severity of PTSD symptoms, and race/ethnicity populations involved in these genetic studies of PTSD^25^.

One way to alleviate the clinical heterogeneity of PTSD is to take advantage of intermediate phenotypes, especially the quantitative neuroimaging phenotypes^26–28^. A growing body of imaging genetics research has proven the association between neuroimaging measures and candidate genes in PTSD^29–31^. Catechol-*O*-methyltransferase (*COMT*) Val^158^Met (rs4680) and brain-derived neurotrophic factor (*BDNF*) Val^66^Met (rs6265) polymorphisms are two typical and well-described genetic variants that have been proven to be associated with stress response and resilience^32–34^. These two genetic variants have been extensively investigated in populations consisting of healthy subjects^33,35^ and those with anxiety disorders^36,37^, including PTSD^29,31^. Meanwhile, both *COMT* rs4680 and *BDNF* rs6265 regulate the brain dopamine system^38^—an important system in the pathogenesis of PTSD—by their important role in regulating fear memory emotion and behaviors. Dopaminergic dysfunction has also been reported to play a role in the arousal symptoms^39^ and fear conditioning to an aversive stimulus^40^, which are seen in PTSD patients. *COMT* catalyzes the degradation of catecholamines, particularly dopamine, and the Met^158^ and Val^158^ alleles of *COMT* rs4680 polymorphism have low and high enzyme activity, respectively^41,42^. *BDNF* is a key neurotrophin that regulates neuronal plasticity, neuronal survival, and neurogenesis, and the Met^66^ allele of *BDNF* rs6265 polymorphism is associated with lower BDNF release compared with the Val^66^ allele, resulting in reduced activity-dependent dopamine release^43^. Both *COMT* rs4680 and *BDNF* rs6265 polymorphisms are suggested to moderate several neuroimaging structural phenotypes observed in PTSD^29,31,44^. In addition, recently, the interaction effect between *COMT* rs4680 and *BDNF* rs6265 on brain function has been reported in healthy subjects^45,46^. However, to the best of our knowledge, no study to date has investigated the effects of *COMT* rs4680, *BDNF* rs6265, or *COMT* by *BDNF* interaction on brain function in PTSD patients. So far, little is known about the effects of *COMT* rs4680 and *BDNF* rs6265 genotypes on brain function in Chinese adults who lost their only child.

In this study, we aimed to examine the main effects of *COMT* rs4680, *BDNF* rs6265, and their interaction effect on brain function as seen using resting-state functional magnetic resonance imaging (fMRI) in Chinese adults who had lost their only child. Degree Centrality (DC)^47,48^—an important fMRI-derived metric that measures the strength of intrinsic connectivity between a given voxel and others in the brain—was used in this study for three reasons. First, DC is a physiologically meaningful and powerful biomarker in exploring the communication potential/strength of a certain brain region^47–50^. Second, the DC algorithm has been successfully applied in studying many mental disorders, such as obsessive-compulsive disorder^51,52^, bipolar disorder^53,54^, and one published fMRI study involving Chinese adults who lost their only child (22 trauma-exposed adults without PTSD diagnosis were recruited in that study)^55^. Third, recent studies using DC have demonstrated modulatory effects of *COMT*^56,57^, *BDNF*^45^, and even *COMT* by *BDNF* interaction^45^ on the connectivity strength of the brain’s functional hubs. In this preliminary study, we hypothesized that *COMT* rs4680 and *BDNF* rs6265 would exhibit different modulatory effects on the DC in prefrontal-limbic regions in Han Chinese adults with loss of an only child and diagnosed with PTSD, when compared with those without PTSD diagnosis.

## Subjects and methods

### Subjects

We performed a PTSD survey in Han Chinese adults who had lost their only child from Jiangsu Province, China, between September 2016 and March 2017. This study was approved by the Medical Research Ethics Committee of Jiangsu University and all participants provided written informed consents. All 237 Han adults (ages 40–67 years, mean age of 58.54 years) who had lost their only child—without other major traumatic exposures detected on the clinician-administered PTSD scale (CAPS) life events checklist—were successfully interviewed and screened by the CAPS. The Chinese version of the structured clinical interview for DSM-IV^58^ (revised by Professor Lipeng Fei from Beijing Hui Long Guan Hospital) was used to screen all these participants. After this procedure, 57 trauma-exposed adults were diagnosed with PTSD (19 out of these 57 PTSD adults had comorbid major depressive disorder (MDD), 3 had comorbid generalized anxiety disorder (GAD), and one had comorbidities of both MDD and GAD). One hundred and seventy trauma-exposed adults did not meet any diagnostic criteria of mental illness or substance-use disorders. Ten trauma-exposed adults were diagnosed with other psychiatric disorders (five diagnosed with MDD, four with GAD, and one with mixed generalized anxiety and MDD) and they were excluded in this study.

The exclusion criteria for the subsequent fMRI study were as follows: any current or history of brain injury or other major medical or neurological conditions (five trauma-exposed adults without PTSD were ruled out: four had cerebral infarction or ischemia and one had a history of MDD and corresponding antidepressant drug therapy), any MRI contraindication (none), and left-handedness (none).

### Measures

Each participant was assessed using a set of neuropsychological tests, which included the Hamilton Depression (HAMD)^59^ and Hamilton Anxiety (HAMA)^60^ rating scales, the Mini-Mental State Examination^61^, Chinese Social Support Rating Scale (SSRS)^62^, and individual Simple Coping Style Questionnaire (SCSQ)^63^. The SSRS contains 3 subscales of social support: subjective support (which reflects the perceived interpersonal network that an individual can count on, 4 items with scores ranging from 8 to 32); objective support (actual support an individual received, 3 items with scores ranging from 1 to 22); and the utility of support (the pattern of behavior that an individual exhibits when seeking social support, 3 items with scores ranging from 3 to 12). Higher scores for the SSRS indicate stronger social support and the total support score (ranging from 12 to 66) is the sum of all the sub-items. The SCSQ contains assessments of both active and negative coping, containing 12 and 8 items, respectively. The scale of each SCSQ item uses 4-level Likert score standards, in which “3” stands for regular use, whereas “0” stands for no use. Next, the scores for active and negative coping are calculated independently, and a higher score indicates the inclination to adopt the corresponding coping style, whereas the coping tendency score is defined as the active coping score minus the negative coping score.

### MRI data acquisition

MRI data were acquired using a 3 Tesla MR scanner (Achieva 3.0 TTX; Philips, Amsterdam, The Netherlands). Foam pads were applied to minimize head motion. The participants were instructed to stay still during scanning and keep their eyes closed but not fall asleep. First, high-resolution T_1_-weighted structural images were acquired with the three-dimensional turbo fast echo sequence (repetition time/echo time [(TR/TE) = 9.7 ms/4.6 ms, field of view (FOV) = 256 × 256 mm^2^, flip angle = 9°, matrix size = 256 × 256, 160 sagittal slices with thickness of 1 mm). Second, rs-fMRI data were acquired with a single-shot, gradient-recalled echo-planar imaging sequence (TR/TE = 2000 ms/30 ms, FOV = 192 × 192 mm^2^, flip angle = 90°, matrix = 64 × 64, voxel size = 3 × 3 × 4 mm^3^). For each subject, 230 brain volumes with 35 axial slices of functional data were collected in 460 s.

### Data preprocessing

MRI data were preprocessed using Data Processing Assistant for Resting-State fMRI (DPARSF; http://rfmri.org/DPARSF)^64^, which is essentially based on SPM12 software (http://www.fil.ion.ucl.ac.uk/spm). The first ten volumes of each subject were excluded for steady-state longitudinal magnetization; the remaining 220 volumes were corrected for temporal differences and head motion. Each subject’s T_1_-weighted image was co-registered to the functional images and then segmented into gray matter, white matter, and cerebrospinal fluid, and then transformed into the standard Montreal Neurological Institute (MNI) space using the Diffeomorphic Anatomical Registration Through Exponentiated Lie algebra. The fMRI data were then transformed into the MNI stereotaxic space of 3 × 3 × 3 mm^3^, using the parameters of the T_1_ image normalization and smoothed with an 8 mm full width at half maximum (FWHM) isotropic Gaussian kernel.

### Quality control and nuisance regression

To address concerns about head motion, according to recent recommendations in minimizing head motion confounds^65,66^, we used the Friston 24-parameter model^67^ to regress out head motion effects. Head translations, rotations, and the framewise displacement (using the Jenkinson formula) from each participant were calculated. We excluded seven participants (two PTSD adults and five trauma-exposed controls) for head translations >1.5 mm or rotations >1.5°, and two participants (trauma-exposed controls) for mean framewise displacement >2.5 SD. We also included the mean framewise displacement as a nuisance covariate in the following imaging genetic analyses. Other sources of spurious variance (mean signals from cerebrospinal fluid and white matter) were also regressed out. The imaging data were also temporally filtered (bandpass: 0.01–0.1 Hz).

After the quality control, 9 subjects were excluded, and 55 PTSD adults and 158 trauma-exposed controls remained.

### Degree centrality analysis

To measure the DC of each voxel throughout the brain, voxel-wise whole-brain correlation analysis with a threshold of *r* = 0.25 was performed^51,54^. We also analyzed the DC with other *r* thresholds (0.2, 0.3, and 0.4, respectively) (please refer to “Validation Analysis” in the Results section for details). The DC of a voxel was calculated as the sum of connectivity between a given voxel and other voxels, which exceeded the *r* threshold^47,48^. For standardization purposes, the DC of each voxel was divided by the global mean DC value.

### DNA genotyping

Of all the subjects enrolled in this study, three trauma-exposed adults without PTSD refused blood collection. DNA for other participants was obtained from peripheral blood samples. Single-nucleotide polymorphism (SNP) genotyping of *COMT* rs4680 and *BDNF* rs6265 (detailed primers were listed in Supplementary Table S1) were conducted using the Improved Multiple Ligase Detection Reaction technique developed by Genesky Biotechnologies, Inc. (Shanghai, China)^68^. About 5% of the samples were randomly selected for confirmation and the results were 100% concordant.

### Statistical analysis

Hardy–Weinberg equilibrium test of *COMT* rs4680 and *BDNF* rs6265 was performed using R version 3.5.3 (https://www.r-project.org). SPSS version 25 (IBM, Corp, Armonk, New York, USA) was used to analyze the clinical and psychological data. A three-way (diagnosis of PTSD, *COMT* rs4680, and *BDNF* rs6265 genotypes) analysis of variance was used to evaluate the main effects of PTSD diagnosis, *COMT* rs4680, *BDNF* rs6265 polymorphisms, and their interaction effects for all the demographic and psychological data. A voxel-wise three-way (diagnosis, *COMT*, and *BDNF*) analysis of covariance (ANCOVA) was performed with SPM12 to assess the individual effect of PTSD diagnosis, *COMT* rs4680, *BDNF* rs6265, and their interaction effects on brain DC map, adjusting for the effects of age, sex, educational level, duration since child-loss trauma, and head motion of framewise displacement. Significant clusters were determined by the Alphasim program, which was implemented in REST1.8 (http://www.restfmri.net/forum/index.php). The threshold was set at combined *P* < 0.005 for each voxel and a cluster size larger than 48 voxels (parameters: FWHM = 8 mm; the number of Monte Carlo simulations = 1000; within the gray matter mask consisting of 67,541 voxels), which corresponds to a corrected *P* < 0.05 (https://afni.nimh.nih.gov/pub/dist/doc/manual/AlphaSim.pdf).

A partial correlation analysis was performed to examine the relationship between DC values of those regions showing significant effects from above ANCOVA and CAPS, SSRS, SCSQ, HAMA, and HAMD, adjusting for age, sex, educational level, duration since child-loss trauma, and head motion. Correlation results were corrected for multiple comparisons using the Bonferroni correction for the number of regions where altered DC was detected from the ANCOVA (cutoff *P*-values of 0.05/10 = 0.005 in this study, corresponding to all ten regions showing significance).

## Results

### Clinical and psychological data

The flowchart of the study population is shown in Supplementary Fig. S1. All 55 PTSD adults and 155 trauma-exposed controls without PTSD were included in the final neuroimaging genetic analyses (Table 1). The distributions of both *COMT* rs4680 (PTSD group: 31 Val/Val, 22 Met/Val, and 2 Met/Met; trauma-exposed control group: 88 Val/Val, 56 Met/Val, and 11 Met/Met) and *BDNF* rs6265 (PTSD group: 17 Val/Val, 27 Met/Val, and 11 Met/Met; control group: 38 Val/Val, 83 Met/Val, and 34 Met/Met) were in Hardy–Weinberg equilibrium (all *P* > 0.05). For each polymorphism, participants were divided into two subgroups (Val-homozygous and Met-allele carriers) based on their genotypes, following methods in previous studies^46,69^ and then grouped based on both *COMT* rs4680 and *BDNF* rs6265 polymorphisms (as detailed in Supplementary Table S2). Among the PTSD samples, there were 8 *COMT* Met-*BDNF* Val/Val, 16 *COMT* Met-*BDNF* Met, 9 *COMT* Val/Val-*BDNF* Val/Val, and 22 *COMT* Val/Val-*BDNF* Met; within trauma-exposed controls, there were 16 *COMT* Met-*BDNF* Val/Val, 51 *COMT* Met-*BDNF* Met, 22 *COMT* Val/Val-*BDNF* Val/Val, and 66 *COMT* Val/Val-*BDNF* Met.

**Table 1:** Demographics and psychological data of Han Chinese adults who lost their only child.

Protocols	Adults with PTSD (*n* = 55)	Adults without PTSD (*n* = 155)	*P*- value
Age (±SD), years	57.56 ± 5.53	58.57 ± 5.54	0.25^a^
Sex (F/M)	39/16	72/83	0.002^b^
Education, years	6.49 ± 4.15	6.68 ± 3.61	0.75^a^
HAMD	15.93 ± 6.71	5.98 ± 4.24	<0.001^a^
HAMA	12.55 ± 6.62	4.66 ± 3.44	<0.001^a^
MMSE	25.84 ± 3.16	26.10 ± 3.33	0.61^a^
Duration since child-loss trauma, month	59.12 ± 48.39	107.46 ± 71.22	0.001^a^
CAPS_total	46.96 ± 12.41	16.63 ± 9.93	<0.001^a^
SSRS			
Objective support	12.40 ± 2.73	12.73 ± 2.67	0.44^a^
Subjective support	21.40 ± 3.88	21.58 ± 3.91	0.77^a^
Utility of support	5.62 ± 2.00	5.55 ± 1.93	0.83^a^
SSRS_total	39.42 ± 7.04	39.86 ± 6.58	0.68^a^
SCSQ			
Active	18.29 ± 6.41	19.58 ± 6.41	0.20^a^
Negative	9.96 ± 2.91	10.38 ± 3.40	0.42^a^
Copying tendency	8.33 ± 5.94	9.24 ± 5.88	0.33^a^

Values are expressed as mean ± SD. *CAPS* clinician-administered PTSD scale, *HAMA* Hamilton Anxiety, *HAMD* Hamilton Depression, *MMSE* Mini-Mental State Examination, *PTSD* posttraumatic stress disorder, *SCSQ* simple coping style questionnaire, *SSRS* social support rating scale.

^a^The *P*-value for the difference between the two trauma-exposed groups was obtained by two sample *t*-test.

^b^The *P*-value for gender distribution between the two trauma-exposed groups was obtained by the *χ*^2^-test.

There were no significant differences between PTSD adults and trauma-exposed controls in age, educational level, SSRS, or SCSQ (*P* > 0.05), but PTSD adults had higher CAPS, HAMA, and HAMD scores, a lower male-to-female ratio, and shorter duration since losing the child (Table 1). There was a significant *COMT* × *BDNF* interaction effect on scores of active coping style (*F* = 4.43, *P* = 0.04) in all participants, irrespective of PTSD diagnosis, which was driven by the lower score in the *COMT* Met-*BDNF* Val/Val subgroup than the *COMT* Met-*BDNF* Met subgroup (*P* = 0.05), whereas no significant difference was found between *COMT* Val/Val-*BDNF* Val/Val and *COMT* Val/Val-*BDNF* Met subgroups (*P* = 0.20). There were no significant *COMT* or *BDNF* main effects, or two-way or three-way interactions for all other clinical and psychological data.

### The main effect of PTSD diagnosis on DC

**Fig. 1:**
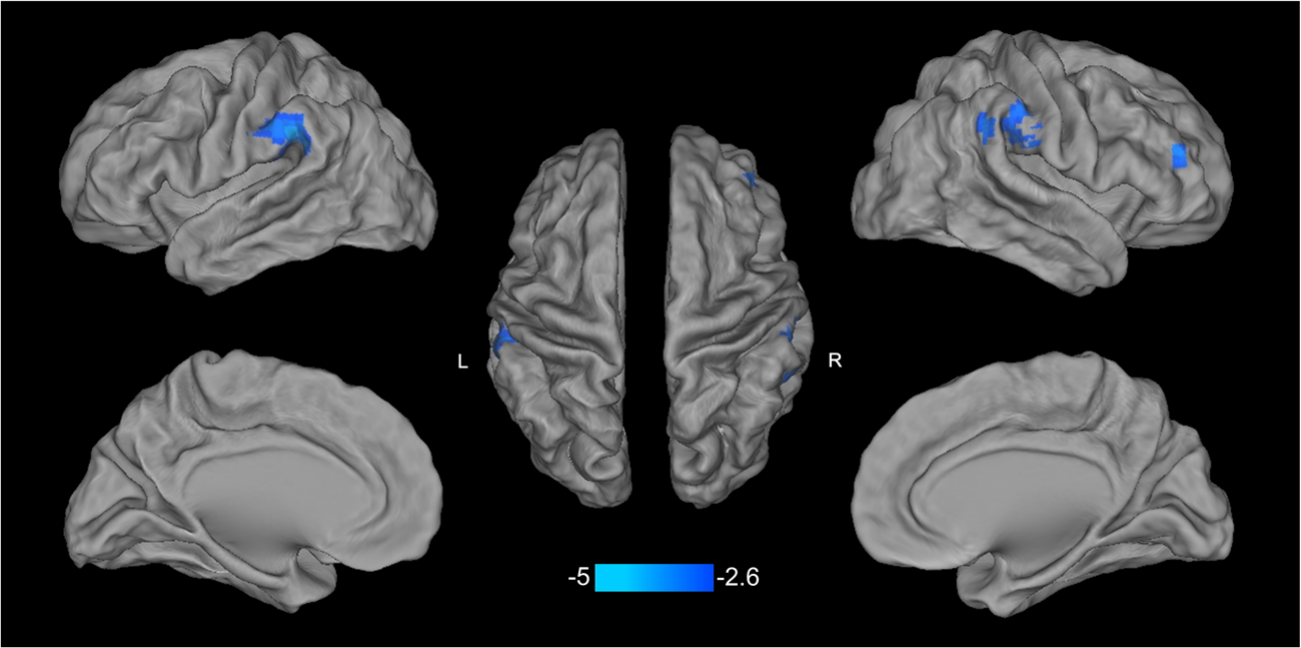
Post-hoc test result of PTSD diagnosis main effect. Post-hoc analysis of PTSD diagnosis main effect shows that PTSD adults have weaker DC in bilateral inferior parietal lobules and right middle frontal gyrus, relative to trauma-exposed adults without PTSD (corrected *P* < 0.05). PTSD posttraumatic stress disorder.

Significant PTSD diagnosis effects on DC were found in bilateral inferior parietal lobules (IPL) and right middle frontal gyrus (MFG). Post-hoc analysis showed that PTSD adults had weaker DC in these three regions, relative to trauma-exposed adults without PTSD (Fig. 1 and Supplementary Table S3).

### The individual effect of *COMT* and *BDNF* genotypes on DC

**Fig. 2:**
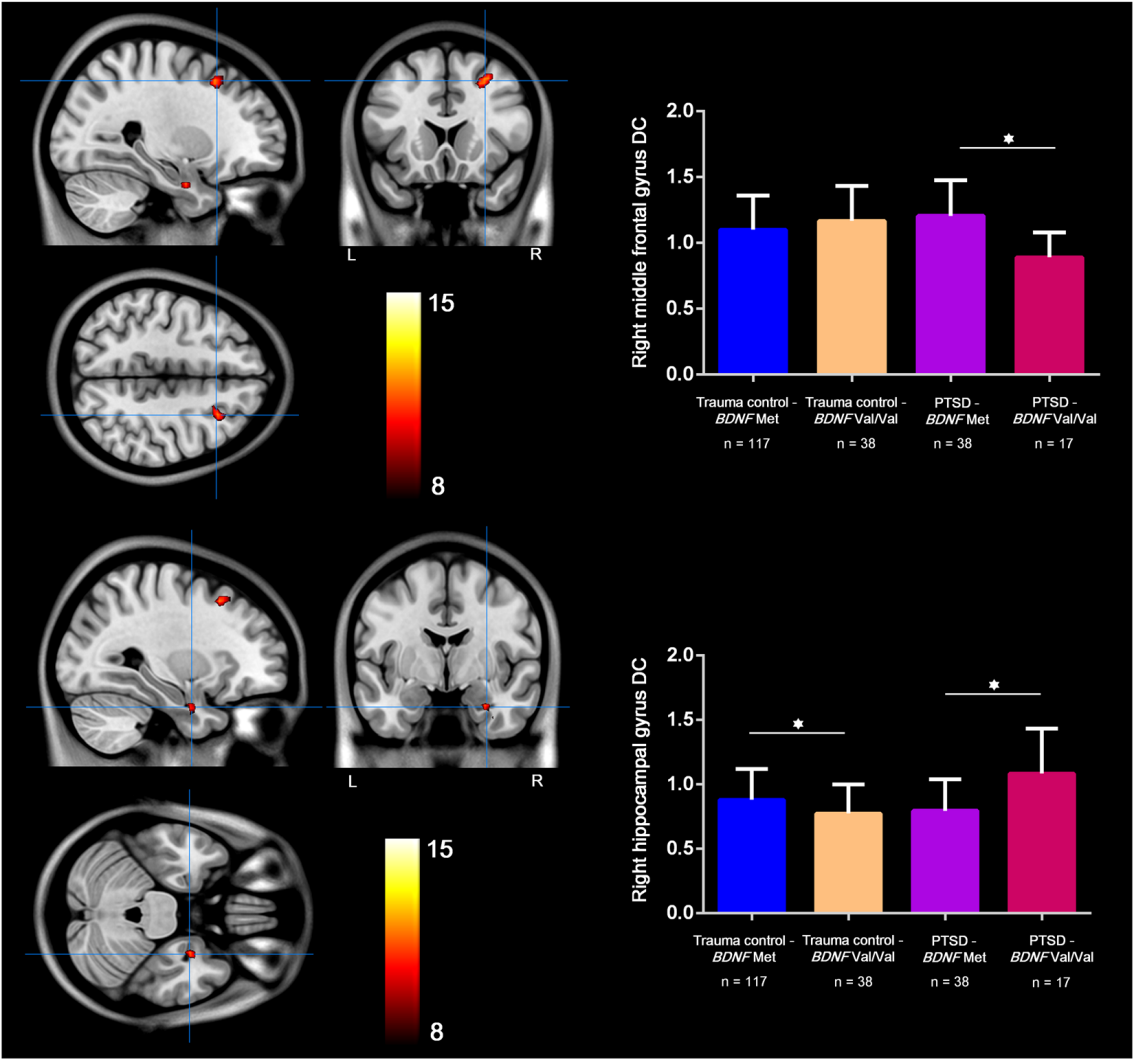
Interaction effect of diagnosis × *BDNF* rs6265 (corrected *P* < 0.05). There are significant *BDNF* genotype × diagnosis interaction effects in the right MFG and marginally significant interaction in the right hippocampus. At the MFG, in PTSD adults, the *BDNF* Val/Val genotype is associated with weaker DC than its Met carrier counterpart (*P* < 0.001). At the hippocampus, in PTSD adults, the *BDNF* Val/Val genotype is associated with higher DC than its Met carrier counterpart (*P* < 0.001), whereas in trauma-exposed controls the opposite occurs (*P* = 0.046). *BDNF* brain-derived neurotrophic factor, *COMT* catechol-*O*-methyltransferase, PTSD posttraumatic stress disorder, SVC small-volume correction.

No significant main effect of *COMT* or *BDNF* genotypes was found in this study. However, there was a significant *BDNF* genotype × diagnosis interaction effect in the right MFG (Fig. 2 and Supplementary Table S3), which accounted for 9% of the interindividual variance in DC in right MFG (partial *η*^2^ = 9%, estimated using SPSS 25). At this locus, in PTSD patients, the *BDNF* Val/Val genotype was associated with weaker DC compared with its Met carrier counterpart (*P* < 0.001), whereas in trauma-exposed controls, there was no significant difference between these two genogroups (*P* = 0.17).

In this study, the right hippocampus—a core region affected by the pathology of PTSD—only passed the *P* < 0.005 threshold but not the cluster size threshold (Supplementary Table S3); we then used a lenient statistical threshold to investigate its potential change^56^. A 20 mm-radius box centered at the peak location of the hippocampus was placed based on the whole brain results of *BDNF* × diagnosis interaction effect (*P* < 0.005), then a family-wise error small-volume correction (SVC), which implemented in SPM12, was conducted to correct for the multiple comparisons within the box^56^. After this procedure, a marginal significance of the right hippocampus was found (*P* = 0.058, SVC corrected). The *BDNF* genotype × diagnosis interaction accounted for 9% variance in DC in this region (*η*^2^ = 9%). Post-hoc analysis showed that at the hippocampus, in PTSD adults, the *BDNF* Val/Val genotype was associated with higher DC than its Met carrier counterpart (*P* < 0.001), whereas in trauma-exposed controls the opposite occurred (*P* = 0.046) (Fig. 2).

### Interaction of *COMT* and *BDNF* genotypes on DC

**Fig. 3:**
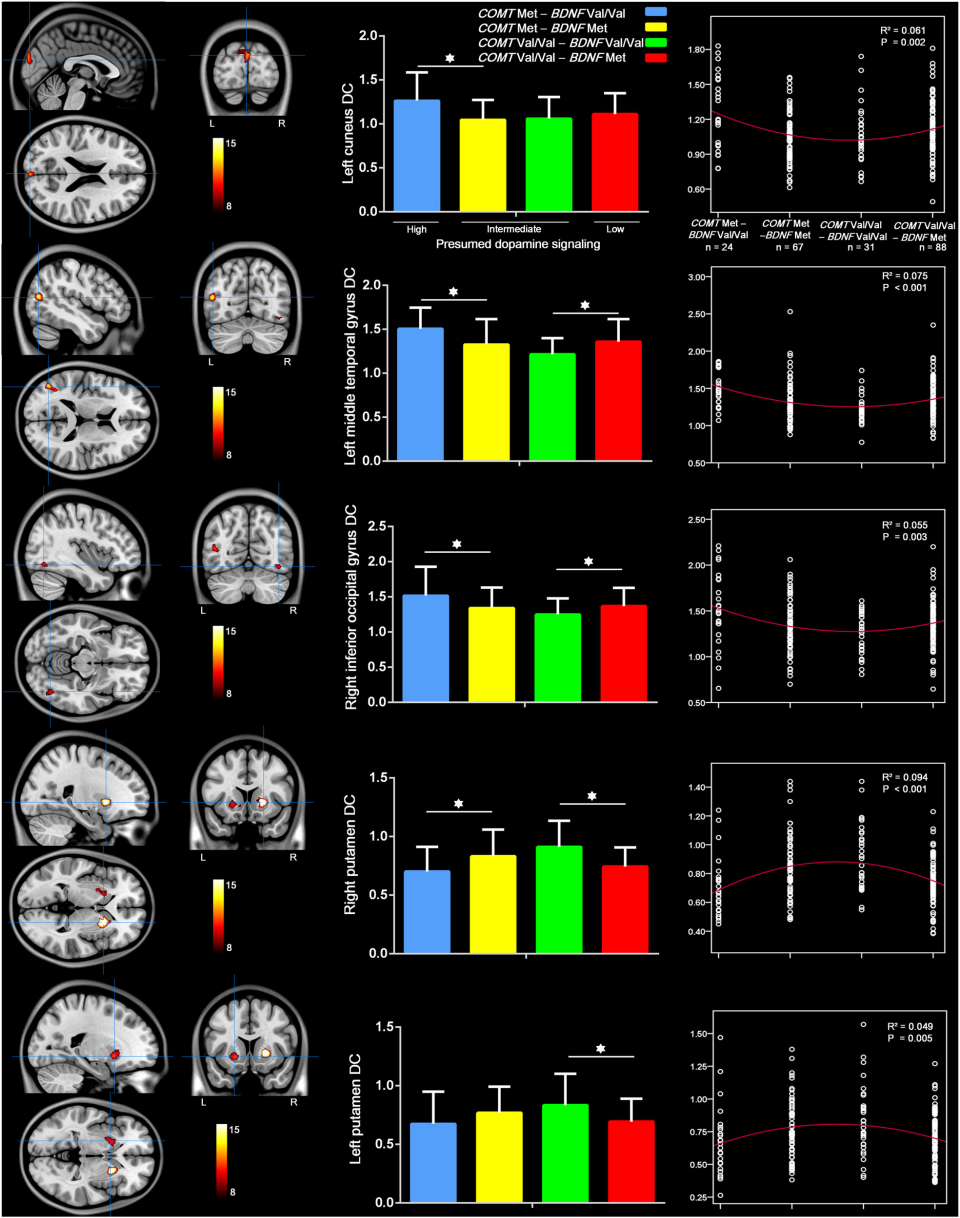
Interaction of *COMT* rs4680 and *BDNF* rs6265 genotypes effect (corrected *P* < 0.05). There are significant interactions between the effects of the *COMT* and *BDNF* genotypes in the left cuneus, left middle temporal gyrus, right inferior occipital gyrus, and bilateral putamen in all participants, irrespective of PTSD diagnosis. The distribution of the DC is likely a U-shaped curve in the cortical regions and an inverted U-shaped curve in the putamen, according to the presumed dopamine signaling from high to low. BDNF brain-derived neurotrophic factor, COMT catechol-*O*-methyltransferase, DC degree centrality, PTSD posttraumatic stress disorder.

There were significant interactions between the effects of the *COMT* and *BDNF* genotypes in the regions of left cuneus (*η*^2^ = 10%), left middle temporal gyrus (*η*^2^ = 11%), right inferior occipital gyrus (*η*^2^ = 10%), and bilateral putamen (right: *η*^2^ = 13%; left: *η*^2^ = 8%) in all participants, irrespective of PTSD diagnosis (Fig. 3 and Supplementary Table S3). Interestingly, after conducting the Curve Estimation procedure with SPSS 25, the quadratic regression was significant in all these regions by sorting according to *COMT* and *BDNF* genotypes (*P* < 0.05). The distribution of the DC was a U-shaped curve in the cortical regions, whereas for the putamen it showed an inverted U-shaped curve (Fig. 3), according to the presumed dopamine signaling from high to low (*COMT* Met-*BDNF* Val/Val genogroup > *COMT* Met-*BDNF* Met and *COMT* Val/Val-*BDNF* Val/Val > *COMT* Val/Val-*BDNF* Met)^45^. Specifically, in the regions of the left cuneus, left middle temporal gyrus, and right inferior occipital gyrus, the DC was higher in the *COMT* Met-*BDNF* Val/Val subgroup than in the *COMT* Met-*BDNF* Met subgroup, but weaker in *COMT* Val/Val-*BDNF* Val/Val than in *COMT* Val/Val-*BDNF* Met (except for the region of the left cuneus, where no significant difference was found between *COMT* Val/Val-*BDNF* Val/Val and *COMT* Val/Val-*BDNF* Met subgroups, with *P* = 0.33). In contrast, for the region of bilateral putamen, the DC was weaker in *COMT* Met-*BDNF* Val/Val than in *COMT* Met-*BDNF* Met subgroups, but higher in *COMT* Val/Val-*BDNF* Val/Val than in *COMT* Val/Val-*BDNF* Met subgroups (in the left putamen, there was no significant difference but a slight trend of *COMT* Met-*BDNF* Val/Val < *COMT* Met-*BDNF* Met, with *P* = 0.08). No significant effect of diagnosis × *COMT* × *BDNF* interaction was found in this study.

### Correlation analysis

**Fig. 4:**
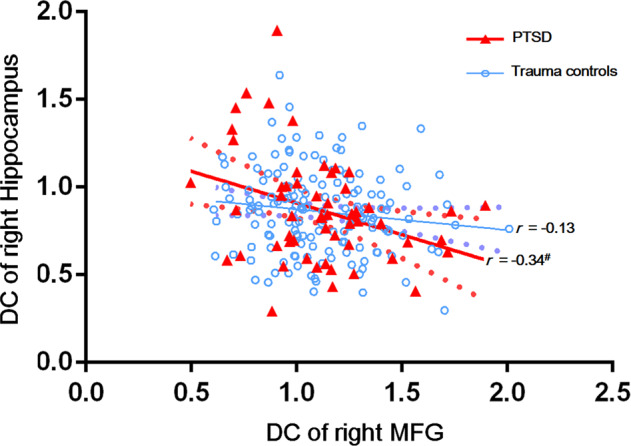
Partial correlation results between the MFG and hippocampus (uncorrected *P* < 0.05). A negative partial correlation (regressed out the age, sex, educational level, duration since child-loss trauma, and head motion) is found between DC in the right hippocampus and right MFG in the PTSD group (*r* = −0.34; *P* = 0.01). ^#^*P* < 0.05. *BDNF* brain-derived neurotrophic factor, DC degree centrality, MFG middle frontal gyrus, PTSD posttraumatic stress disorder.

For regions showing significant effect of diagnosis × *BDNF* interaction, a negative partial correlation was found between the right hippocampus and right MFG DC values, only in the PTSD group (*r* = −0.34; *P* = 0.01; Fig. 4). However, after multiple testing correction, it was no longer statistically significant. No significant correlation was found between regions showing significant PTSD main effect, *COMT* × *BDNF* interaction, and clinical or psychological indices.

### Validation analysis

We assessed the validity of our findings through several strategies: first, we used different correlation coefficient thresholds for the DC measurement, with *r*-values = 0.2, 0.3, and 0.4, respectively. Similar results were found, except for the loss of significance of *COMT* × BDNF interaction effect in the region of inferior occipital gyrus at *r* = 0.3 and 0.4, after the same multiple comparisons correction. Second, as PTSD adults showed higher HAMA and HAMD scores than those without PTSD, we also performed the statistical analysis by additionally including anxiety and depression (along with gender and age, education, duration, and head motion) as covariates to evaluate the effect of anxiety and depression on the results. We found that some regions’ effects became insignificant after the multiple comparisons (Supplementary Table S3). This finding may suggest that part of the changes could be accounted for by a higher level of anxiety and depression, which was in line with the frequently observed accompanying depression and anxiety symptoms in PTSD subjects in actual clinical practice^70,71^. Third, to assess the possible effect of volume differences in detected regions, we extracted regional gray matter volume of each region from our findings and compared each of them with a similar three-way ANCOVA, and found no significant effects on gray matter volume of these regions (a detailed description was provided in the Supplementary Note 1). Fourth, for head motion control, despite concerns that scrubbing could compromise graph construction for DC analysis by increasing the likelihood of extreme correlation values, we conducted the scrubbing (removing time points with framewise displacement >0.2 mm)—along with the same strategy at the analyses to verify the results. All results remained the same after using this aggressive head motion control strategy. Furthermore, to ensure that our results are specific to the effects of *BDNF* and *COMT* rather than other dopamine-related genes, we further included five other dopamine-related SNPs—two *DRD2* SNPs (rs2075652 and rs2134655)^72^ and three *DRD3* SNPs (rs4646996, rs7131056, and rs9868039)^73^—in a multiple liner regression model to investigate their effects on the brain regions from our current results (SNP main effect and SNP × diagnosis interaction effect). After adopting the Bonferroni correction threshold (*P* = 0.005), we did not find any statistically significant effects of these five dopamine SNPs (a detailed description was provided in the Supplementary Note 2).

## Discussion

In this study, we investigated the effects of PTSD diagnosis, *COMT* rs4680, and *BDNF* rs6265 polymorphisms on brain functional connectivity strength in Han Chinese adults who had lost their only child. We found the main effect of PTSD diagnosis on DC in the regions of IPL and MFG, and the interaction effect of *BDNF* × diagnosis in MFG and hippocampus. In addition, there was significant *COMT* × *BDNF* interaction effect in regions of the left cuneus, left middle temporal gyrus, right inferior occipital gyrus, and bilateral putamen in all participants, irrespective of PTSD diagnosis.

Converging evidence has suggested prefrontal-limbic imbalance along with a hypoactive (prefrontal)–hyperactive (limbic) gradient in PTSD^12^. For example, an increasing number of neuroimaging studies^74,75^ and meta-analytic reviews^12,76^ consistently demonstrated decreased activity in prefrontal regions and increased activity in limbic regions in PTSD patients. In supplement to prefrontal-limbic imbalance, the cognitive-affective imbalance theory^8,77^ was also propounded, where brain executive system (under-activated) and emotional processing system (over-activated) are differentially affected by PTSD. The IPL and dorsolateral prefrontal cortex (DLPFC, lies within the MFG) are two core parts of the brain executive-control network^78^ and both of them have been demonstrated to be susceptible to the effects of stress, partially due to the fact that these two regions developmentally mature later than other regions^79,80^. Decreased IPL or DLPFC activity was often reported in adults with a history of early life stress exposure^79,81^ and patients with PTSD^77,82^. Besides, decreased connectivity strength in IPL and DLPFC were detected by Liu et al.^55^ in a small group of Chinese adults who lost their only child (compared with healthy controls without trauma exposure). Thus, the weaker DC in IPL and MFG observed in the current study aligns with the findings of prior studies and provides further insight into understanding the changes of their communication potential/strength when PTSD occurs in bereaved adults.

Besides, we found an interaction effect of *BDNF* × diagnosis in the present study. As for MFG, the *BDNF* Val/Val genotype was associated with weaker DC than its Met carrier counterpart in PTSD adults, but for trauma-exposed controls, there was an opposing trend. A nonlinear, inverted U-shaped relationship between dopamine levels with spatial working memory and related cortical (prefrontal) function has been robustly demonstrated by a series of studies, both in non-human primates^83^ and humans^84^. This inverted U-shaped curve predicts that too much or too little dopamine can impair cortical function, and this has been applied to investigate the disruption of brain function in several mental disorders^85–87^. For example, Prata et al.^85^ reported that the cortical efficiency in patients with schizophrenia may shift to the left limb of the inverted U-shaped curve. In Parkinson’s disease, the attention function and prefrontal function tended to shift to the right limb of the curve (based on putative prefrontal DA levels)^87^. In healthy controls, the *BDNF* Met polymorphism was thought to be associated with higher cognitive deficit risk and lower frontal activity, especially within the context of working memory^88^. Dopamine plays a crucial role in neurological processes such as reward, motivation, and stress^89^. Increasing evidence has demonstrated dopaminergic hyperactivity as an underlying mechanism in PTSD-related pathophysiology^90^. Single-photon emission computed tomography study also found increased dopamine transporter density in bilateral striatum in PTSD patients^91^, which was interpreted as a contributing factor to the perpetuation and potentiation of exaggerated fear responses to a particular event associated with the traumatic experience. In the current study, as the increased putative dopamine signaling in *BDNF* Val/Val genotype was associated with decreased MFG DC in the PTSD group, we speculate that the inverted U-shaped relationship between dopamine signaling and frontal function may shift to the right limb in these bereaved PTSD adults (Supplementary Fig. S2). Although a few studies reported that the *BDNF* Met allele may be related to increased PTSD susceptibility^92^, other studies^93^ and recent reviews^94,95^ did not support this relationship. A precise understanding of the role of *BDNF* rs6265 polymorphism on PTSD development is required in further studies.

Another interesting finding in this study was that the interaction effect of *BDNF* × diagnosis in the hippocampus was almost opposite to the MFG, where the *BDNF* Val/Val genotype was associated with higher hippocampal DC compared with its Met carrier counterpart in PTSD adults but reversed in trauma-exposed controls. Besides, the DC values of MFG and hippocampus were anticorrelated in the PTSD group. As the hippocampus here was just marginally significant after multiple testing corrections, we explain the underlying biological mechanisms with caution. However, several explanations could be proposed based on current knowledge. The hippocampus is essential for memory functions, especially to memorize facts and events, and memory consolidation^96^. A series of studies have demonstrated greater hippocampal engagement in response to negative stimuli and trauma-specific cues, but less engagement during exposure to positive images^97^. Prefrontal-limbic imbalance in PTSD is thought to result in the failure to inhibit the negative memory^8,12^. Here we speculate a potential U-shaped relationship between the presumed dopamine levels and hippocampus function (Supplementary Fig. S2), in which both lower dopamine in trauma-exposed controls and higher dopamine in PTSD adults would be associated with lower hippocampal efficiency. This is shown as increased hippocampal DC, which may be related to overgeneralized relevance to negative stimuli and trauma-specific cues (may indicate inefficiency/disturbance of hippocampal connectivity)^12^. This speculation was supported by a study in a large number of healthy subjects, in which the *BDNF* Val/Val genotype subgroup had higher hippocampal activity than the Met carrier genotype subgroup during a working memory task, but accomplished with poorer episodic memory^43^. However, this speculation needs to be validated with a larger sample and more studies in the future.

In the present study, *COMT* × *BDNF* interaction effects were found in several temporal and occipital regions and putamen, irrespective of PTSD diagnosis. Besides, the distribution of the DC is more likely to be a U-shaped curve in these cortical regions and an inverted U-shaped curve in the putamen, according to the presumed dopamine signaling from high to low. The U-shaped and inverted U-shaped curves modulated by dopamine signaling have recently been reported in several studies^45,98,99^. For example, Zhao et al.^98^ reported network-dependent modulations of dopamine signaling on brain function, in which the intra-network functional connectivities of the brain “processing system” and “control system” were differently modulated by the *COMT* × *DRD2* interaction effects. Based on current substantiated knowledge that the prefrontal and striatal dopamine systems in the brain have an antagonistic relationship, particularly in pathological circumstances^100,101^, we propose that there might be an anti-relationship of brain dopamine modulation in cortical regions and basal ganglia regions. A future study that includes both trauma-exposed subjects and non-traumatized healthy controls would be suitable to validate whether this phenomenon is unique in trauma-exposed subjects.

This study has several limitations. First, our study only focused on the influence of losing an only child, a unique phenomenon accompanying One-Child Policy in China, and so we urge caution when generalizing these resulting interpretations to other traumatic events and other race/ethnicity populations. Second, the cross-sectional nature of our measurement did not allow us to ascertain whether the abnormalities in PTSD and the effect of gene modulation were present before the traumatic experience, or if they occurred after the traumatic event. Third, we note that several other genes—such as dopamine transporter and receptor genes—involved in dopamine functioning^102^ should be addressed in future studies to clarify the modulatory effects of dopamine pathway genes on brain function^103^. Finally, given the history of inconsistent replication of candidate-gene studies^104,105^, the findings in this study should be considered preliminary and need to be validated by studies using large replication samples or using data from GWAS of PTSD^19,106^.

In conclusion, our findings provide evidence to suggest that the modulatory effect of BDNF polymorphism on the MFG and hippocampus may contribute to PTSD development in bereaved adults. Interactions of *COMT* × *BDNF* polymorphisms modulate some cortices and basal ganglia, irrespective of PTSD development. In future, tests of interventions and treatment effects in patients with PTSD may benefit from stratifying patients into groups by genotype, or at least from modeling genotype effects on brain metrics that may differ by genotype. Eventually, there may be sufficient data to test for treatment × genotype interaction effects on brain phenotypes relevant to PTSD, a key goal of precision medicine.

## Supplementary information

Supplementary Material
