# Supplementary material for: Effects of COMT rs4680 and BDNF rs6265 polymorphisms on brain degree centrality in Han Chinese adults who lost their only child
Source: Transl Psychiatry. 2020 Jan 30;10:46. doi: 10.1038/s41398-020-0728-7 (PMC7026113; doi:10.1038/s41398-020-0728-7)
Supplement: Supplementary file 1 — Supplementary Material [file 41398_2020_728_MOESM1_ESM.docx]

**Effects of COMT rs4680 and BDNF rs6265 Polymorphisms on Brain Degree Centrality in Han Chinese Adults who Lost Their Only Child**

**Supplementary Material**

1. **Supplementary Note 1: Validation analysis for possible confounding effect of brain structural changes**
2. **Supplementary Note 2: Validation analysis for possible confounding effect of other dopamine genes**
3. **Supplementary Figures**
4. **Supplementary Tables**
5. **Supplementary Note 1: Validation analysis for possible confounding effect of brain structural changes**

To assess whether the functional results in the present study might be confounded by regional brain structural changes, we further performed voxel-based morphometry (VBM) to examine possible structural deficits, by using the CAT12 Toolbox (http://dbm.neuro.uni-jena.de/cat12/). We applied the default settings according to the manual of CAT12 (<http://dbm.neuro.uni-jena.de/cat12/CAT12-Manual.pdf>) except for using the affine regularization using the International Consortium for Brain Mapping template for East Asian brains. The individual T_1_-weighted images underwent bias-correction, tissue classification, and were transformed into standard MNI space. Then the individual T_1_-weighted images were segmented into gray matter, white matter and cerebrospinal fluid. The segmented gray matter images were smoothed with an FWHM of 8 mm. We extracted regional gray matter volume of each region from our findings and compared each of them with a similar three-way ANCOVA. No any significant *COMT* or *BDNF* main effects, or any other two-way or three-way interactions were found on the gray matter volumes of all these regions.

1. **Supplementary Note 2: Validation analysis for possible confounding effect of other dopamine genes**

To ensure that our results are specific to the effects of *BDNF* and *COMT* rather than other dopamine genes, we further included five other dopamine-related SNPs – two *DRD2* SNPs (rs2075652 and rs2134655)^1^ and three *DRD3* SNPs (rs4646996, rs7131056, and rs9868039)^2^ – in a multiple linear regression model to investigate their effects on the brain regions from our current results (SNP main effect and SNP × diagnosis interaction effect). The A allele of these five dopamine SNPs has previously been reported to be correlated with greater risk of PTSD diagnosis^1, 2^, so for each subject, the SNP genotype was coded as a covariate based on whether each individual carried the A risk allele (non A allele carriers = 1, A allele carriers=2). The distributions of each dopamine SNP genotype were as follows:

*DRD2* rs2075652 – PTSD group with 27 non A allele carriers and 28 A allele carriers, control group with 53 non A allele carriers and 102 A allele carriers;

*DRD2* rs2134655 – PTSD group with 31 non A allele carriers and 24 A allele carriers, control group with 76 non A allele carriers and 79 A allele carriers;

*DRD3* rs4646996 – PTSD group with 7 non A allele carriers and 48 A allele carriers, control group with 27 non A allele carriers and 128 A allele carriers;

*DRD3* rs7131056 – PTSD group with 26 non A allele carriers and 29 A allele carriers, control group with 50 non A allele carriers and 105 A allele carriers; and

*DRD3* rs9868039 – PTSD group with 17 non A allele carriers and 38 A allele carriers, control group with 48 non A allele carriers and 107 A allele carriers.

The regression model we used was as follows:

Regional DC value = *β_0_* + *β_1_* SNP + *β_2_* diagnosis + *β_3_* SNP genotype × diagnosis +*β_4_* age + *β_5_* sex + *β_6_* education + *β_7_* duration since trauma + *β_8_* head motion + *ε*,

– with a significance threshold set at corrected *P* < 0.05 (cut-off *p* values of 0.05/10 = 0.005 – corresponding to a total of 10 brain regions in the current results). We only found that the *DRD2* rs2134655 polymorphism had a marginally significant main effect on degree centrality in the left cuneus (*P* = 0.047, *β= -0.16*), and the *DRD3* rs9868039 polymorphism had a marginally significant main effect in the right putamen (*P* = 0.04, *β= 0.17*). Also, there was a marginally significant *DRD3* rs9868039 polymorphism × diagnosis interaction effect in the right inferior occipital gyrus (*P* = 0.018, *β= 0.627*). But after adopting the Bonferroni correction threshold (*P* = 0.005), none of these results remained statistically significant. Therefore we could infer that our current results mainly resulted from the effects of *COMT* and *BDNF* rather than other dopamine genes (in the scope of the five dopamine SNPs we tested here).


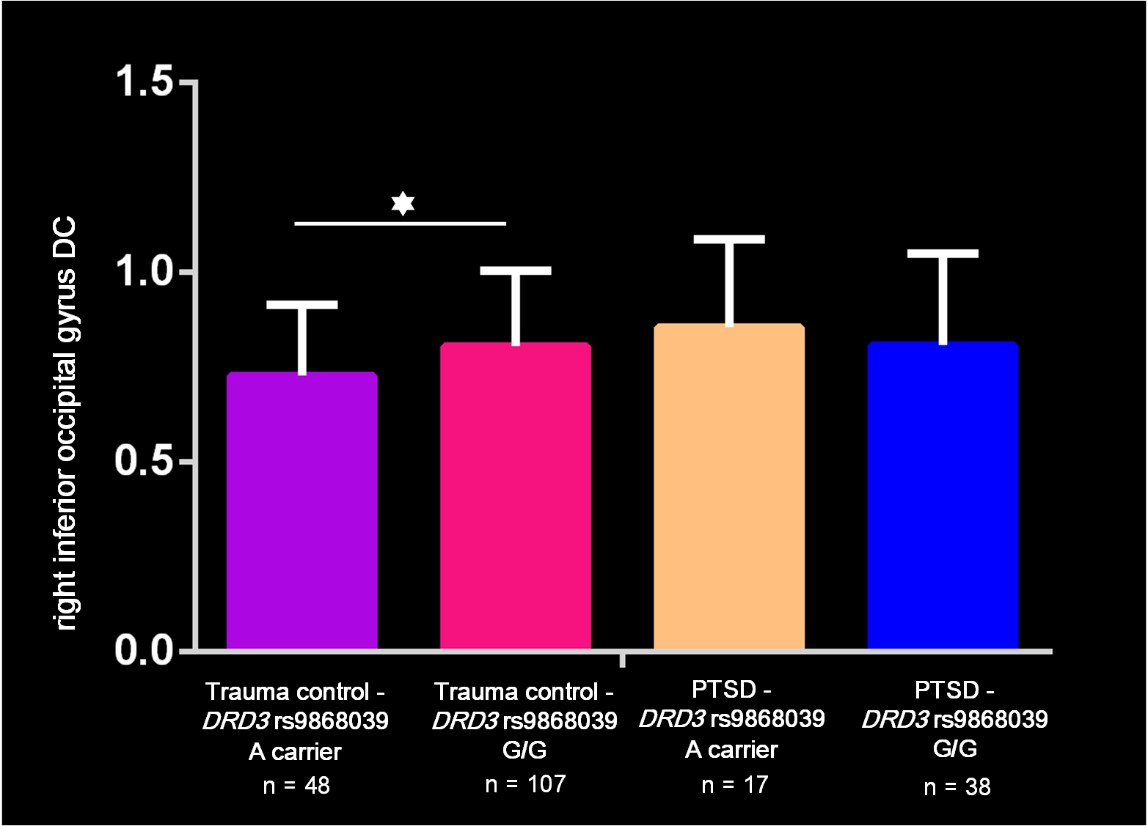


**Interaction effect of diagnosis × *DRD3* rs9868039 (*uncorrected *P* < 0.05).**

There was a nominally significant *DRD3* rs9868039 polymorphism × diagnosis interaction effect on degree centrality (DC) in the right inferior occipital gyrus (*P* = 0.018, *β= 0.627*). In trauma-exposed controls, *DRD3* rs9868039 A genotype is associated with weaker DC than the G/G genotype (*P* = 0.033), whereas, in PTSD adults, there was no difference between the two subgroups.

1. **Supplementary Figures**

**Figure S1.** The flowchart of the study population in the study.

**
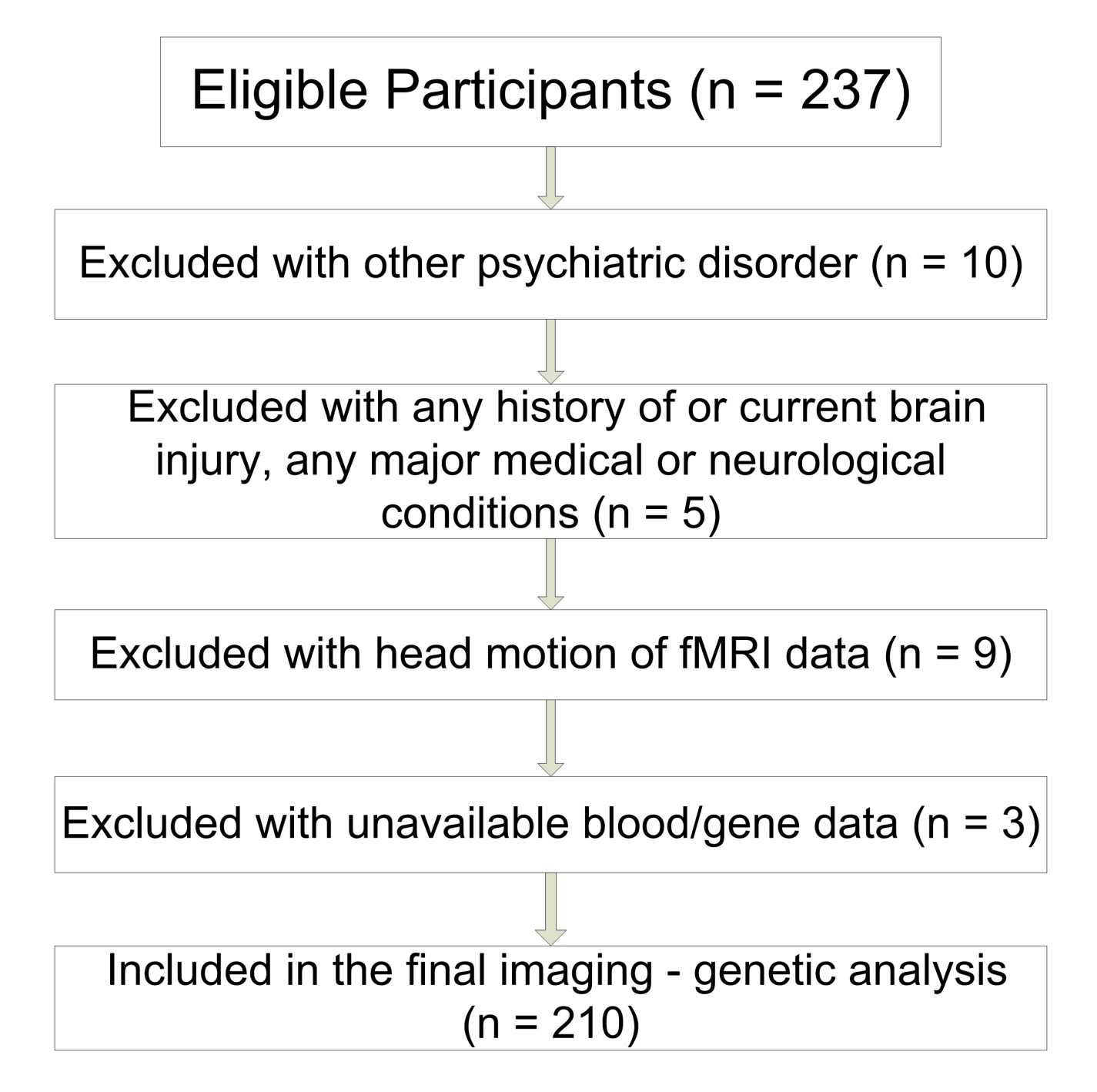
**

**Figure S2.** Putative relationship between dopamine signaling and frontal, hippocampal function in bereaved adults with and without PTSD.


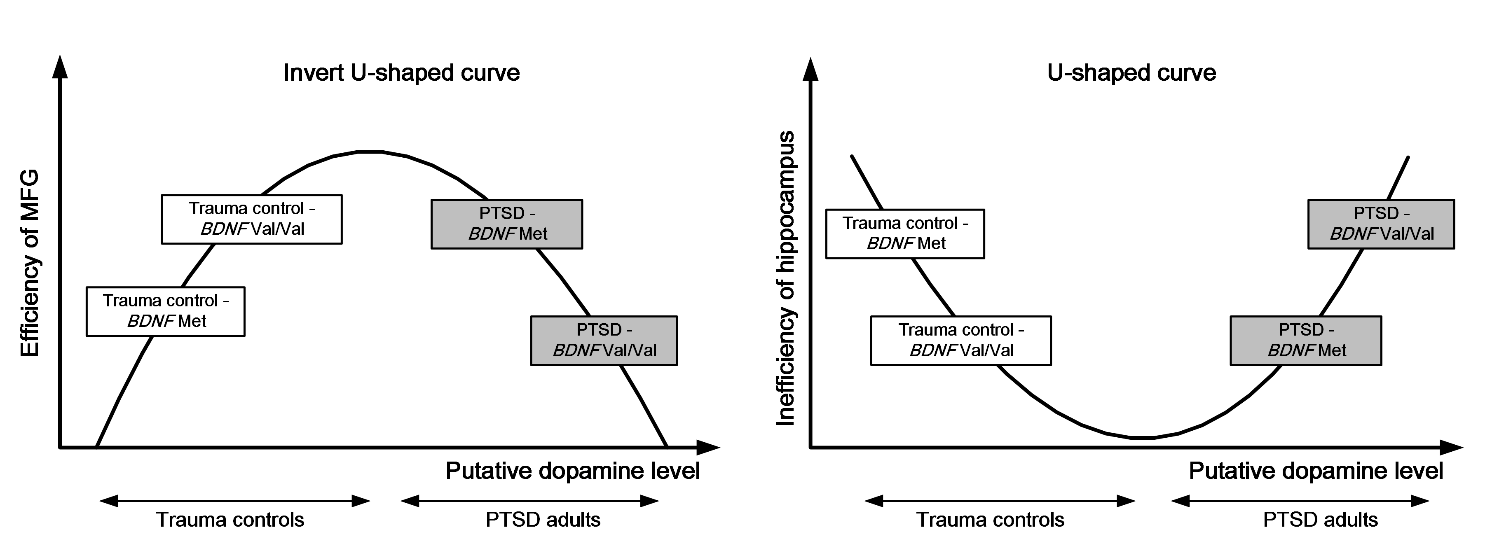


Results from our findings support the theory that too much or too little brain dopamine levels are associated with reduced prefrontal efficiency (inverted U-shaped curve in MFG). We further find that the MFG and hippocampus are reversely modulated by the interaction effect of *BDNF* genotype and diagnosis, suggesting that there might be different modulation relationships in these two regions (inverted U-shaped curve in MFG and U-shaped curve in hippocampus, respectively).

*BDNF* = brain-derived neurotrophic factor; PTSD = post-traumatic stress disorder; MFG = middle frontal gyrus.

1. **Supplementary Tables**

**Table S1.** Primer sequences for the *COMT* and *BDNF* SNPs

| SNP | Chromosome position | PCR Primer |  |
| --- | --- | --- | --- |
| rs4680 | 19951271^a^ | Forward | AAGATCGTGGACGCCGTGATT |
|  |  | Reverse | ACACCTGGTGGGGAGGACAAAG |
| rs6265 | 27679916^a^ | Forward | TGAGCATCACCCTGGACGTGTA |
|  |  | Reverse | TGGGACTCTGGAGAGCGTGAAT |

^a^ Reference Genome: hg19

*COMT* = Catechol-O-methyltransferase; *BDNF* = brain-derived neurotrophic factor; SNP = single nucleotide polymorphism; PCR = Polymerase chain reaction.

| **Table S2: Clinical and psychological data of *COMT* rs4680 and *BDNF* rs6265 genotypes in Han Chinese adults who lost their only child with and without PTSD** | Adults PTSD (N=55) | | | | | Adults without PTSD (N=155) | | | | |
| --- | --- | --- | --- | --- | --- | --- | --- | --- | --- | --- |
| Protocols | *COMT* Met- *BDNF* Val/Val (N=8) | *COMT* Met -*BDNF* Met (N=16) | *COMT* Val/Val -*BDNF* Val/Val (N=9) | *COMT* Val/Val-BDNF Met (N=22) | *P* value | *COMT* Met -*BDNF* Val/Val (N=16) | *COMT* Met -*BDNF* Met (N=51) | *COMT* Val/Val -*BDNF* Val/Val (N=22) | *COMT* Val/Val -*BDNF* Met (N=66) | *P* value |
| Age (±SD), y | 59.50±3.02 | 57.31±6.29 | 56.44±4.39 | 57.50±6.15 | 0.72^a^ | 60.25±4.73 | 57.94±5.96 | 59.91±4.81 | 58.21±5.58 | 0.30^a^ |
| Sex (F/M) | 6/2 | 11/5 | 7/2 | 15/7 | 0.94^b^ | 6/10 | 20/31 | 10/12 | 36/30 | 0.34^b^ |
| Education, y | 7.50±3.21 | 7.31±4.09 | 6.00±4.74 | 5.73±4.33 | 0.59^a^ | 6.00±3.63 | 6.59±3.65 | 6.95±3.26 | 6.82±3.74 | 0.85 ^a^ |
| HAMD | 17.00±7.61 | 15.38±7.43 | 15.22±5.54 | 16.23±6.66 | 0.93^a^ | 6.73±3.95 | 6.14±4.24 | 4.41±3.89 | 6.23±4.39 | 0.30^a^ |
| HAMA | 14.13±9.89 | 11.56±4.90 | 12.44±5.25 | 12.73±7.11 | 0.85^a^ | 5.67±3.79 | 4.65±3.58 | 3.18±2.84 | 4.94±3.36 | 0.12^a^ |
| MMSE | 26.12±2.10 | 26.13±3.10 | 24.67±3.87 | 26.00±3.31 | 0.70^a^ | 24.63±6.64 | 26.24±2.38 | 26.32±3.29 | 26.28±2.77 | 0.32^a^ |
| Duration since child-loss trauma, month | 69.63±49.00 | 60.06±45.31 | 89.11±75.40 | 42.36±29.42 | 0.09^a^ | 117.25±81.64 | 93.37±62.10 | 101.82±62.93 | 117.85±77.02 | 0.28^a^ |
| **CAPS** |  |  |  |  |  |  |  |  |  |  |
| CAPS_total | 45.00±11.49 | 43.56±11.36 | 49.33±5.98 | 49.18±15.10 | 0.50^a^ | 17.93±8.08 | 15.45±10.67 | 18.00±8.42 | 16.79±10.26 | 0.71^a^ |
| **SSRS** |  |  |  |  |  |  |  |  |  |  |
| Objective support | 12.25±2.60 | 12.56±2.92 | 13.44±2.07 | 11.91±2.91 | 0.56^a^ | 13.13±2.33 | 12.71±2.81 | 12.18±2.40 | 12.83±2.76 | 0.72^a^ |
| Subjective support | 21.63±3.34 | 21.63±3.34 | 22.06±4.47 | 21.04±4.37 | 0.74^a^ | 21.62±1.71 | 22.20±3.86 | 20.50±5.00 | 21.44±3.96 | 0.40^a^ |
| Utility of support | 5.25±1.91 | 5.50±2.13 | 6.56±2.07 | 5.45±1.92 | 0.49^a^ | 4.38±1.82 | 5.92±1.96 | 5.14±1.64 | 5.69±1.93 | 0.03^a^ |
| SSRS_total | 39.13±6.33 | 39.13±6.33 | 42.66±7.63 | 38.41±7.41 | 0.50^a^ | 39.13±4.26 | 40.82±6.65 | 37.82±7.96 | 39.97±6.44 | 0.33^a^ |
| **SCSQ** |  |  |  |  |  |  |  |  |  |  |
| Active | 16.13±4.02 | 19.69±7.39 | 19.44±6.69 | 17.59±6.32 | 0.54^a^ | 18.40±5.08 | 20.90±5.36 | 20.14±6.94 | 18.61±7.15 | 0.23^a^ |
| Negative | 10.63±1.06 | 11.13±3.00 | 10.44±1.24 | 8.68±3.37 | 0.054^a^ | 10.63±3.96 | 10.80±3.62 | 10.50±2.32 | 9.94±3.39 | 0.58^a^ |
| Copying tendency | 5.50±4.34 | 8.56±5.85 | 9.00±7.42 | 8.91±5.96 | 0.55^a^ | 8.25±5.16 | 10.10±5.66 | 9.64±6.62 | 8.67±6.00 | 0.53^a^ |

Values are expressed as mean ± SD. *COMT* = Catechol-O-methyltransferase; *BDNF* = brain-derived neurotrophic factor; PTSD = post-traumatic stress disorder; HAMD = Hamilton Depression; HAMA = Hamilton Anxiety; MMSE = Mini-Mental State Examination; CAPS = clinician-administered PTSD scale; SSRS = social support rating scale; SCSQ = simple coping style questionnaire.

^a^ The *P* value for the difference among the four genogroups was obtained by one-way analysis of variance test.

^b^ The *P* value for gender distribution among the four genogroups was obtained by the chi-square test.

**Table S3: The effects of PTSD diagnosis, *COMT*, and *BDNF* on DC in Han Chinese adults who lost their only child.**

|  | Results included age, sex, education, duration, and head motion as covariates | | | | Anxiety added | Depression added | Anxiety and depression added |
| --- | --- | --- | --- | --- | --- | --- | --- |
| Brain regions | BA | x, y, z | *F* value | Voxel (N) | Voxel (N) | Voxel (N) | Voxel (N) |
| **Group main effect** |  |  |  |  |  |  |  |
| IPL, left | 40 | -57,-42,33 | 17.82 | 123 | 53 | 143 | 124 |
| IPL, right | 40 | 60,-39,33 | 14.16 | 115 |  |  |  |
| MFG, right | 10 | 42,48,18 | 14.91 | 51 |  | 48 |  |
| **Group** × ***BDNF* interaction effect** |  |  |  |  |  |  |  |
| MFG, right | 38 | 27,18,48 | 12.05 | 50 | 52 | 52 | 54 |
| Hippocampus, right | 8 | 27,-3,-27 | 11.72 | 24^a^ | 36^b^ | 26^a^ | 36^b^ |
| ***COMT*** × ***BDNF* interaction effect** |  |  |  |  |  |  |  |
| Cuneus, left | 19 | 0,-93,24 | 13.78 | 82 |  | 66 |  |
| MTG, left | 39 | -48,-72,15 | 14.83 | 61 |  |  |  |
| IOG, right | 19 | 39,-66,-12 | 10.86 | 49 |  |  |  |
| Putamen, right |  | 21,12,0 | 21.07 | 97 | 60 | 73 | 60 |
| Putamen, left |  | -21,12,-6 | 11.32 | 80 | 107 | 122 | 107 |

PTSD = post-traumatic stress disorder; *COMT* = Catechol-O-methyltransferase; *BDNF* = brain-derived neurotrophic factor; DC = degree centrality; BA = Brodmann area; IPL= inferior parietal lobules; MFG = middle frontal gyrus; MTG = middle temporal gyrus; IOG = inferior occipital gyrus.

^a^ The *P* value is marginally significant after small volume correction.

^b^ The *P* value ˂ 0.05 after small volume correction.

**REFERENCES**

1. Duan Z*, et al*. Assessment of functional tag single nucleotide polymorphisms within the DRD2 gene as risk factors for post-traumatic stress disorder in the Han Chinese population. *J Affect Disord* 2015; **188:** 210-217.

2. Wolf EJ*, et al*. The dopamine D3 receptor gene and posttraumatic stress disorder. *J Trauma Stress* 2014; **27**(4)**:** 379-387.
